# A systematic review and analysis of long-term outcomes in attention deficit hyperactivity disorder: effects of treatment and non-treatment

**DOI:** 10.1186/1741-7015-10-99

**Published:** 2012-09-04

**Authors:** Monica Shaw, Paul Hodgkins, Hervé Caci, Susan Young, Jennifer Kahle, Alisa G Woods, L Eugene Arnold

**Affiliations:** 1 Clinical Development & Medical Affairs, Shire Pharmaceuticals, Ltd, Basingstoke, UK; 2 Global Health Economics & Outcomes Research, Shire Development LLC, 725 Chesterbrook Boulevard, Wayne, PA, 19087, USA; 3 Child and Adolescent Psychiatry Hôpitaux Pédiatriques de Nice CHU Lenval, 57, Avenue de la Californie, F-06200 Nice, France; 4 King's College London, Institute of Psychiatry, De Crespigny Park, London, SE5 8AF, UK; 5 BPS International, 3830 Valley Centre #705 PMB503, San Diego, CA 92130, USA; 6 Biochemistry and Proteomics Laboratory, Chemistry and Biomolecular Science, Science Center, Room 158, Clarkson University, Potsdam, New York, 13699, USA; 7 Research Unit on Pediatric Psychopharmacology, 207 McCampbell Hall, Ohio State University, Ohio, 43219-1257, USA

**Keywords:** ADHD, adult, childhood, outcomes, psychiatry, systematic

## Abstract

**Background:**

In childhood, attention deficit/hyperactivity disorder (ADHD) is characterized by age-inappropriate levels of inattentiveness/disorganization, hyperactivity/impulsiveness, or a combination thereof. Although the criteria for ADHD are well defined, the long-term consequences in adults and children need to be more comprehensively understood and quantified. We conducted a systematic review evaluating the long-term outcomes (defined as 2 years or more) of ADHD with the goal of identifying long-term outcomes and the impact that any treatment (pharmacological, non-pharmacological, or multimodal) has on ADHD long-term outcomes.

**Methods:**

Studies were identified using predefined search criteria and 12 databases. Studies included were peer-reviewed, primary studies of ADHD long-term outcomes published between January 1980 to December 2010. Inclusion was agreed on by two independent researchers on review of abstracts or full text. Published statistical comparison of outcome results were summarized as poorer than, similar to, or improved versus comparators, and quantified as percentage comparisons of these categories.

**Results:**

Outcomes from 351 studies were grouped into 9 major categories: academic, antisocial behavior, driving, non-medicinal drug use/addictive behavior, obesity, occupation, services use, self-esteem, and social function outcomes. The following broad trends emerged: (1) without treatment, people with ADHD had poorer long-term outcomes in all categories compared with people without ADHD, and (2) treatment for ADHD improved long-term outcomes compared with untreated ADHD, although not usually to normal levels. Only English-language papers were searched and databases may have omitted relevant studies.

**Conclusions:**

This systematic review provides a synthesis of studies of ADHD long-term outcomes. Current treatments may reduce the negative impact that untreated ADHD has on life functioning, but does not usually 'normalize' the recipients.

## Background

In childhood, attention deficit/hyperactivity disorder (ADHD) is a psychiatric condition characterized by age-inappropriate levels of inattention, hyperactivity-impulsiveness or a combination of these problems [1,2]. The symptoms of ADHD often lead to functional impairment in multiple domains and lower quality of life. Therefore, in recent years the focus of intervention has expanded from ameliorating immediate symptoms of ADHD to improving functionality in several life domains. Moreover, although traditionally regarded as a childhood disorder, it is now clear that ADHD affects both children and adults. The worldwide prevalence of ADHD has been estimated at 5.29% [3,4] with approximately 4% prevalence in adults [5,6]. According to one meta-analysis, ADHD persists in about 65% of adults diagnosed as children if ADHD in partial remission is included [7], and in about 50% of adults originally diagnosed as children according to a separate estimate [8]. Persistence of ADHD may be related to ADHD symptom severity, number of symptoms, ADHD symptom subtype, ADHD in relatives, psychosocial adversity, psychiatric comorbidities, and/or parental psychopathology [8-15]. Many adults with ADHD are undiagnosed and untreated. Research on ADHD in adulthood is relatively sparse [16] despite being recognized in adults as early as 1968 as 'minimal brain dysfunction' [17] and in 1972 as 'hyperkinetic disorder' [18]. Thus the negative outcomes reported by most follow-up studies may be a consequence of untreated symptoms.

The short-term effect of ADHD treatment on symptoms is well characterized. Beyond this, the longer-term consequences have been the focus of numerous individual studies but comprehensive synthesis of the available data has yet to be conducted, thus the present systematic review was performed, focusing on comprehensive summary of long-term outcomes of ADHD. Short-term studies have demonstrated decreases in core symptoms with pharmacotherapy, but there is less evidence for longer-term benefits. Poor adherence and persistence on therapy, comorbidities, poor follow-up and difficulty in accessing consistent medication management from the healthcare system may contribute to difficulty in measuring long-term effects of medication [19,20]. Non-pharmacological interventions such as specialized training for parents of children with ADHD and cognitive behavioral therapy (CBT) for adults also reduce symptoms, and a multimodal approach may have greater effect [16,21]. Both non-pharmacological (that is, psychological, social, and educational) and pharmacological treatments for ADHD are recommended by the National Institute for Health and Clinical Excellence (NICE) guidelines [22] with treatment selection depending on the age of the individual and ADHD severity. Recently published European adult guidelines for the treatment of ADHD indicate that both medications and non-pharmacological interventions may be effective for adults with ADHD, although more research specifically in adults is needed [23].

The importance of long-term studies has been recognized for more than a decade [24]. ADHD treatment guidelines as well as conclusions drawn by health technology assessment agencies recognize that ADHD is potentially a lifelong condition with a profound effect on quality-of-life [22,25-32]. Several of these organizations recognize a need for further study of the long-term consequences of ADHD and of its treatment [22,29,30,32,33]. For example, the NICE guidelines state that 'More research is needed on the influences on eventual outcome, and should include enquiry about the possible benefits (and risks) of early diagnosis and treatment' [22]. Guidelines from the Oregon Health and Science University propose that 'Good-quality evidence on the use of drugs to affect outcomes relating to global academic performance, consequences of risky behaviors, social achievements, etc. is lacking'. [34]. Because many studies of long-term outcomes (LTOs) have in fact been conducted [35], these statements may be more reflective of the quality and variability of data, rather than a lack of ADHD LTO studies. Comprehensive analysis of all available data would therefore be of value, and such an analysis is the purpose of the present review.

The National Institute of Health in the US funded the Multimodal Treatment Study of Children with ADHD (MTA); this is one of the largest independent trials examining the effects on ADHD symptoms and long-term outcomes of different ADHD treatments including: intensive behavioral intervention, medication, these two treatments combined, or routine community care [36]. In the primary intent-to-treat analyses, outcomes in this study were not significantly different for pharmacological treatment alone versus combined treatment after correction for multiple tests, but the combined group allowed as good a result with significantly lower dose of medication (methylphenidate). In two secondary analyses compositing several outcome measures, combined treatment was significantly better than pharmacological treatment alone [36-38]. An 8-year follow-up for this trial failed to differentiate the four treatment groups, demonstrating that regardless of treatment, participants showed improved outcomes (delinquency rating, reading and overall academic performance, and social skills) compared with baseline (pretreatment) [39]. The reasons why the original differences between groups disappeared after 8 years has been extensively debated, with arguments on opposite sides that medication was no longer effective or that all participants improved from treatment and the improvement was sustained or that the natural course of the disorder accounted for the improvement. The best interpretation may be that the data were confounded and conclusions difficult to draw [39-41]. The MTA study is the best-known study of the long-term outcomes of ADHD, including the early impact of treatment on later outcomes.

### Rationale

Based on the potential for long-term persistence of ADHD into adulthood and equivocal reports on treatment, we initiated this evidence-based systematic review to understand the long-term outcomes of ADHD with emphasis on a comprehensive synthesis of published data. Given the large differences in study design and measurements used, we decided that using a binary feature common to all the studies ('significantly different' and 'not significantly different') to define outcome results for LTOs would allow us to summarize all the included studies in a non-statistical fashion. Binary variables are used to simplify data in clinical trials for ADHD quite frequently (for example, Clinical Global Impression-Improvement (CGI-I), ADHD Rating Scale (ADHD-RS) responder analyses). 'Long term' was defined as 2 years or more and 'outcomes' were defined as life consequences, distinct from symptoms.

### Objectives

Our analysis sought to answer the research question: what are the long-term outcomes in participants with ADHD compared to baseline or controls and do long-term outcomes of ADHD improve with treatment (including pharmacological, non-pharmacological and multimodal)?

## Methods

Studies included in this review examined outcomes of (1)participants with untreated ADHD, and (2) participants with treated ADHD. Studies that only examined symptoms (as opposed to life-consequence outcomes) were excluded. The dataset comprised studies published between January 1980 to December 2010, including longitudinal studies with prospective follow-up or retrospective measures of 2 years or more; cross-sectional studies comparing two ages differing by 2 years or more; and single cross-sectional studies of participants age 10 years or older. Age 10 was chosen as the age limit in single cross-sectional studies, based on the *Diagnostic and Statistical Manual of Mental Disorders *fourth edition, text revision (DSM-IV-TR) diagnosis criteria that symptoms be present before age 7 years. Examining outcomes at age 10 years or older, would conservatively allow at least 2 years to pass before outcomes were assessed, in line with our definition of 'long term' as 2 years or more. All studies were peer-reviewed, primary research articles in the English language with full text available. Studies for which all participants were less than 2 years old were excluded. Meta-analyses, case studies, and literature reviews were excluded.

The search methods for identification of studies are summarized below. More specific details of the search methods are provided in Additional File 1. The following inclusionary terms and subterms were included: (1) names of the condition; attention deficit disorder (captured all versions of ADHD), hyperkinesis, TDAH (trouble déficit de l'attention/hyperactivité in French, or trastorno por déficit de atención con hiperactividad in Spanish), DAH (déficit de l'attention/hyperactivité in French, or déficit de atención con hiperactividad in Spanish), DAA (déficit de l'attention/activité in French, or déficit de atención y actividad in Spanish), (2) long-term outcomes; long-term, longitudinal, education, degree, socioeconomic, salary, divorce, relationship, hobbies, criminality, arrest, incarceration, automobile, car, driving, citation, weight, obesity, suicide, drug abuse, addiction, substance abuse, alcoholism, and (3) comparator condition or group; control, proband, placebo, untreated, no treatment, pretreatment, comparator, follow-up, normal. The following exclusionary terms were included: (1) developmental, causal, or symptom as subject terms (not general text words); neuroanatomy, neuropathology, molecular, gene, development, etiology, preclinical, dose-finding, reaction time, and (2) publication types; reprint, review, conference presentation.

The country of origin of each study was noted. For some analyses, studies were grouped by world region (Northern America and the Rest of the World, as defined by the United Nations GeoScheme). Studies from Northern America included those from Canada and the USA. Northern America was identified as a comparator because of the high percentage of participants who are treated for ADHD in these two countries.

To assess risk of bias, we considered search bias, researcher bias, bias of individual research groups and bias due to changes in diagnostic criteria over time.

In the analysis of outcome results, outcomes were considered different between study groups if they were reported to be statistically significantly different in the study or were presented by the study authors as obviously different so as to not require statistical comparison (for example, a study in Norway found that 80% of the study sample with ADHD were unemployed, while the unemployment rate in Norway was 3.5% at the time.) Outcomes that were not statistically significantly different were considered 'similar' to the comparator. We summarized number of outcomes as one measurement and number of studies as a separate measurement, because some studies reported more than one outcome. A list of all the studies included in the final analysis is detailed in a separate publication [35].

## Results

### Data collection and analysis

Our search method has been described in a previous publication [35]. To identify as many published studies as possible, 12 databases were searched: Academic Search Premier, CINAHL, Cochrane CRCT (including EMBASE), Criminal Justice Abstracts, ERIC, MEDLINE, Military & Government collection, NHS Economic Evaluation database, PsycARTICLES, PsycINFO, SocINDEX, and Teacher Reference Center. MEDLINE was searched using two different search engines. Duplicates were eliminated electronically and manually, yielding 5,467 studies.

Based primarily on title and abstract, these studies were reviewed manually and inclusion was agreed on by two researchers. This yielded 351 studies for inclusion in the analysis. A list of all studies included in the analysis has been published [35].

All disagreements between researchers on study inclusion were resolved by examining the full text of the study. Studies included participants who were diagnosed with ADHD or symptomatic presentation of ADHD as reported by the authors of each study. Only those studies in which ADHD was the primary disorder under study were included. Studies included both naturalistic examination of ADHD course (vs non-ADHD controls or start-of-study baseline) and/or treated ADHD (vs ADHD natural course, pretreatment baseline, or non-ADHD controls). Treatments included pharmacological, non-pharmacological, and/or multimodal treatment.

Data from each study was manually extracted from the full text of the study to a database, including: (1) study location, (2) study sample size, (3) study length, (4) participant age range, (5) study support, (6) diagnostic criteria, (7) study type (longitudinal, cross-sectional, prospective, retrospective), (8) outcome measures, (9) outcome results, (10) comparator type, (11) treatment type, and (12) treatment duration. Outcome results were measured by dichotomizing all results into a binary variable of either 'poorer' or 'similar' outcomes, and with respect to outcomes with treatment, either 'benefit' or 'no benefit' with treatment. Many studies (44%) reported more than one outcome result, thus the number of outcome results is more than the number of studies. While the outcomes may be the item of interest, the number of studies from which these outcomes are derived is also informative and so is also reported.

### Outcome groups

**Figure 1:**
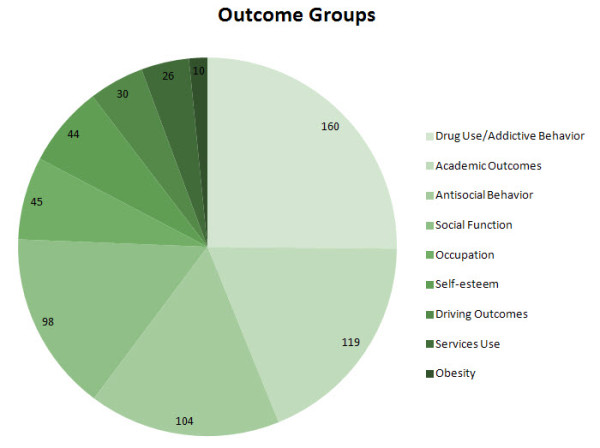
**Number of outcome results by group**. The pie chart shows the number of outcome results by outcome group. Note that the number of outcomes exceeds the number of studies included, because some studies examined more than one outcome. The greatest number of outcomes was measured for drug use/addictive behavior, followed by academic, antisocial behavior, social function, occupation, self-esteem, driving, services use, and obesity outcomes.

Outcome measures were compiled into nine major groups based on commonality of outcome characteristics (Figure 1). This grouping of outcomes has also been described in a prior publication [35]. These groups included: (1) non-medicinal drug use/addictive behavior (for example, use, abuse, and dependence on alcohol, cigarettes, marijuana, stimulants, or illicit drugs; age at first use; multiple substance use; gambling), (2) academic (for example, achievement test scores, grade point average, repeated grades, years of schooling, degrees earned), (3) antisocial behavior (for example, school expulsion, delinquency, self-reported crimes, arrests, detainment, incarceration, repeat convictions), (4) social function (for example, relationships, peer nomination scores, marital status, multiple divorces, activities, hobbies), (5) occupation (for example, employment, military service, job changes, occupation level, socioeconomic status), (6) self-esteem (for example, self-esteem scales, self-perception, suicide ideation, suicide attempts, suicide rate) (7) driving (for example, accidents, traffic violations, license suspensions, driving record), (8) services use (for example, justice system, emergency health care, financial assistance), and (9) obesity (body mass index (BMI), weight).

### Outcome result frequency

The number of outcome results in each outcome group can be seen in Figure 1. Drug use/addictive behavior was the most-studied outcome (160 outcome results), followed by academic (119 outcome results), antisocial behavior (104 outcome results), social function (98 outcome results), occupation (45 outcome results) self-esteem (44 outcome results), driving (30 outcome results), and services use (26 outcome results). Obesity was the least studied outcome (ten outcome results). Note that the total number of outcomes results (636) is greater than the total number of studies (351) because some studies reported more than a single outcome result.

**Figure 2:**
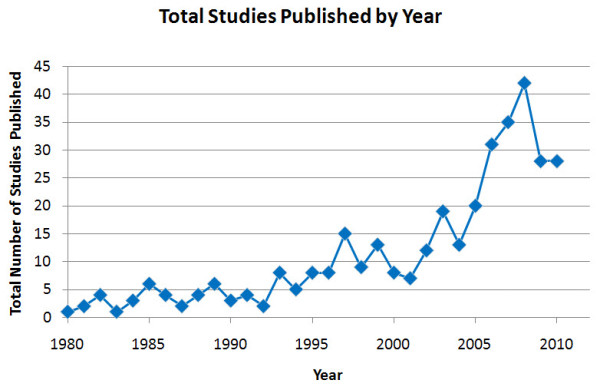
**Total number of studies of long-term outcomes of attention deficit hyperactivity disorder (ADHD) published by year**.

Figure 2 shows the total number of studies published per year. There was a noticeable rise in studies of long-term outcomes of ADHD published worldwide between 1980 and 2008. The number of long-term outcome studies published at the peak in 2008 was 42 studies, dropping back to 28 in 2009 and 2010. The mean study length varied little by year, with a total mean of 9 years for which researchers collected data for each subject and range of 2 to 40 years for which researchers collected data for each subject. Data collection refers either to follow-up measures in the case of prospective studies or analysis of past records or reports in the case of retrospective studies.

### Participant ages

**Figure 3:**
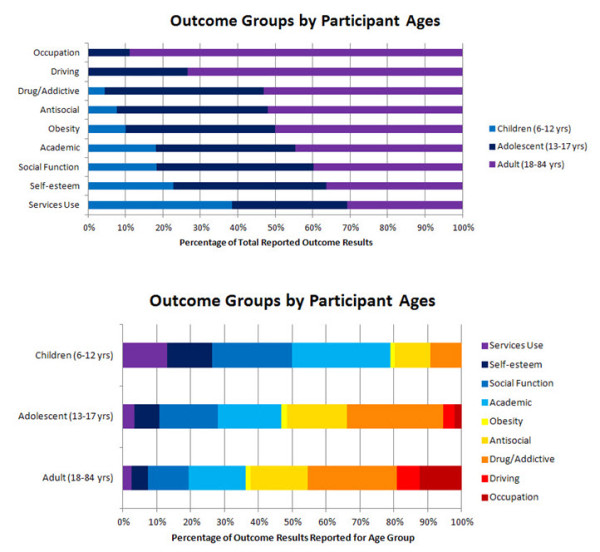
**Outcome groups by ages**. **(A) **This graph shows the mid-range/mean ages of the participants measured in studies of specific outcomes. The light blue portion of the bars represent children 6 to 12 years old, the dark blue bars represent adolescents (13 to 17 years) and the violet bars represent adults (18 to 84 years). The greatest proportion of outcomes examined in children can be seen on the bottom (services use), whereas a greater proportion of outcomes examined in adults can be seen on the top (occupation). **(B) **This graph shows the proportion of outcomes reported within each age category. Each colored section corresponds to the outcomes reported for each outcome group as a proportion of the total number of outcomes reported for that age category.

The ages of the participants were examined by studies of specific outcomes (Figure 3A). Studies of children with a mid-range or mean age of 6 to 12 years measured services use, self-esteem, social function, academic outcomes, obesity, antisocial behavior, and drug use/addictive behavior, in that order of frequency. All nine outcome groups were measured in adults and adolescents. Within age categories (Figure 3B), social function and academic outcomes comprised the largest proportion of children outcomes (53%), while drug use/addictive behavior and antisocial behavior comprised the largest proportion of adult and adolescent outcomes (43 and 46%, respectively). A substantial proportion of outcomes in children and adolescents together were self-esteem and social function outcomes (28%).

### Outcomes with untreated ADHD

**Figure 4:**
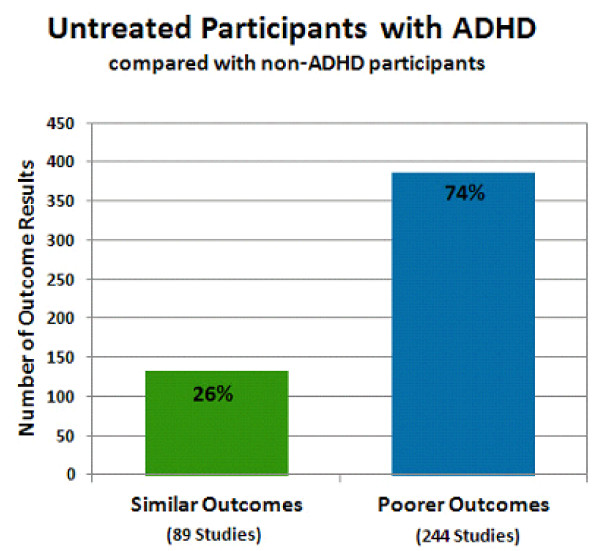
**Untreated participants with attention deficit hyperactivity disorder (ADHD) compared with non-ADHD controls**. The percentage of the total number of outcomes is provided for each bar. The total number of studies represented in each bar is shown in parentheses. The green bar shows the percentage of outcome results reported as similar (26% of outcomes; 89 studies) in untreated participants with ADHD compared with non-ADHD participants. The blue bar shows the percentage of outcome results reported as poorer (74% of outcomes; 244 studies) in untreated participants with ADHD compared with non-ADHD participants. The sum of the numbers of studies shown under each bar does not equal the total number of studies represented in this figure; several studies reported some outcomes that were similar to control and some outcomes that were poorer than controls. Therefore these studies are represented in both types of outcome.

Poorer outcomes were generally observed in untreated participants with ADHD (Figure 4). In all, 89 studies showed that people with untreated ADHD had outcomes not substantially different from controls (26% of outcome results), whereas 244 studies showed that untreated participants with ADHD experienced poorer long-term outcomes (74% of outcome results). Note that more outcomes were observed than studies because some studies reported more than one outcome. There were a few studies (6) that reported outcomes (6) for participants with untreated ADHD that were significantly better than non-ADHD controls. The derived or reported effect sizes were not large, and these few outcomes were included in the outcomes that were 'similar' to controls. No single outcome group was represented; the outcomes varied among drug use/addictive behavior, occupation, self-esteem, and social function outcomes.

Also found in the search were five studies that followed untreated participants with ADHD over 2 to 9 years and compared the long-term outcomes with the participants' status at baseline [42-46]. Four of these studies reported a significant deterioration from baseline without treatment [42-45]. Of these four, three reported a deterioration in academic outcomes (increased number of failing grades over 2 years and decline in math and reading scores over 9 years) [42-44] and one [45] reported a worsening in tobacco use outcomes (levels of salivary cotinine measured daily rose in untreated participants with ADHD over 2 years. Cotinine is an alkaloid from tobacco and a metabolite from nicotine, used as a measure of the number of tobacco cigarettes smoked per day). One study followed untreated participants with ADHD for 8 years into adolescence and reported an improvement in social function compared with the participant's baseline measured at the beginning of the study, although function remained significantly poorer than non-ADHD control levels [46].

Many studies did not report effect size, and effect sizes varied among studies that did report it. For example, a study of driving outcomes reported a small effect size with a Cohen's *d *of 0.33 (*P *= 0.04) for the difference in the number of traffic accidents in the last 6 months for participants with ADHD (0.29 ± 0.73 accidents) compared with non-ADHD controls (0.15 ± 0.43 accidents), albeit this small effect size represented almost twice the rate of accidents for the ADHD group [47]. Another study of social function reported a large effect size with a Cohen's *d *of 1.03 (*P *< 0.001) for the difference in the parent-reported peer rejection scores for children with ADHD (0.45 ± 0.55) compared with non-ADHD controls (0.07 ± 0.23), while controlling for conduct disorder as a comorbidity [48]. It is possible in studies of smaller sample size that there may have been small effects that were not reported as differences, because statistical significance was not demonstrable due to the small sample size. For the purposes of the present analysis, to provide an overall comprehensive synthesis of reported study results, all results were analyzed as reported without additional interpretive changes on our part. Inclusion in this regard was limited by the report having passed through the peer-review process.

### Outcomes with ADHD treatment

**Figure 5:**
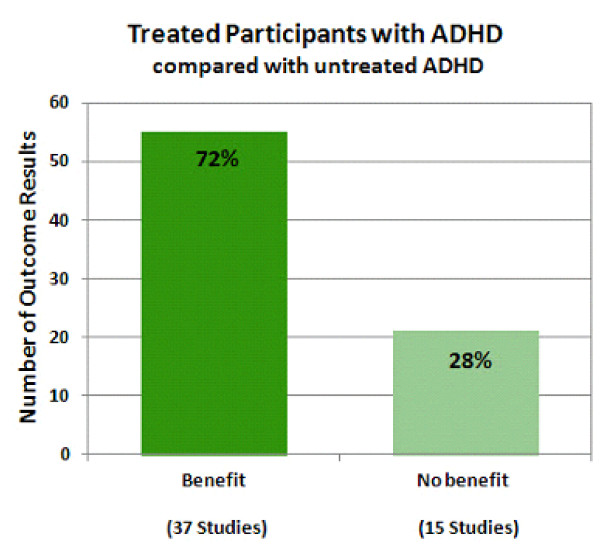
**Treated participants with attention deficit hyperactivity disorder (ADHD) compared with untreated ADHD**. The percentage of the total number of outcome results is provided for each bar. The total number of studies represented in each bar is shown in parentheses. The dark green bar shows the percentage of outcome results reported as exhibiting benefit (72% of outcomes; 37 studies) in treated participants with ADHD compared with untreated ADHD. The light green bar shows the percentage of outcome results reported as exhibiting no benefit (28% of outcomes; 15 studies) in treated participants with ADHD compared with untreated ADHD. Similar to Figure 4, the sum of the numbers of studies shown under each bar does not equal the total number of studies of this type, because several studies have reported some outcomes that exhibited benefit from treatment and some that did not and so these studies are represented in both types of outcome.

Treated ADHD versus untreated ADHD was compared in 48 studies with 76 outcomes (Figure 5). 'Untreated ADHD' comparators included both pretreatment baseline comparisons and comparison with an untreated group of participants with ADHD. Overall, treatment of ADHD resulted in favorable outcomes for most outcomes reported (55 of 76 outcome results; 72%). Three types of outcome results for which treatment was considered beneficial were: (1) improvement compared with participants with untreated ADHD (38% outcome results), (2) improvement compared with pretreatment baseline (22% outcome results), and (3) stabilization compared with pretreatment baseline (12% outcome results). Stabilization compared to pretreatment baseline was considered a benefit of treatment because it indicated that treatment may have alleviated the natural-course deterioration in outcomes over time that has been observed in separate study samples of untreated ADHD [42,43,45,46]. None of the studies reporting stabilization compared with pretreatment baseline also included an untreated ADHD group, therefore this is an across-study sample comparison, subject to limitations. Three types of outcome results for which there was considered no benefit with treatment were: (1) no difference compared with participants with untreated ADHD (25% outcome results), (2) poorer outcomes compared with participants with untreated ADHD (1.5% outcomes results), and (3) poorer outcomes compared with pretreatment baseline (1.5% outcomes results). In outcomes for which there was no difference compared with participants with untreated ADHD, while there was no benefit to treatment, there was also no detriment either, such as increased incidence of substance use disorder or increased rate of suicide. No significantly increased incidence of substance abuse disorders or suicide rate compared with participants with untreated ADHD (or compared with pretreatment baseline either) was reported in any study included in this analysis. There was a single outcome following treatment that was worse for participants with untreated ADHD (increased experimentation with cocaine). A single outcome was reported to be worse than pretreatment baseline (greater percentage of study participants with decreased grade point average).

In 42 studies, the outcome results (n = 76) of participants with treated ADHD were compared with the outcomes of non-ADHD controls. Again, more outcomes were observed than studies because some studies reported more than one outcome. Most such studies did not show normalization with treatment. Only 18 outcomes in 16 studies were similar for participants with treated ADHD versus non-ADHD controls. A total of 58 outcomes (76% of outcomes) in 35 studies, poorer outcomes were observed for participants with treated ADHD relative to non-ADHD controls.

**Figure 6:**
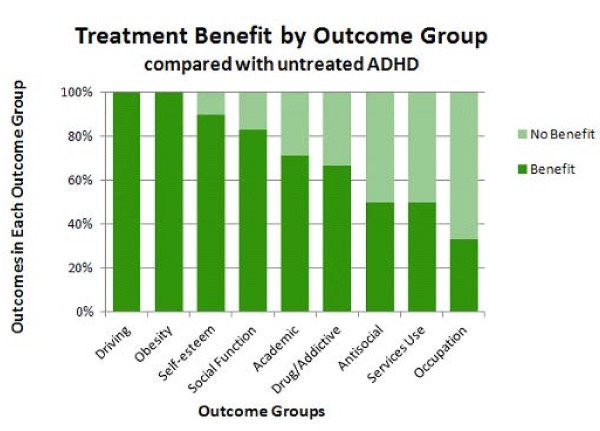
**Benefit and no benefit with treatment by outcome group**. This graph shows benefit (dark green bars) or no benefit (light green bars) by outcome group in treated participants with attention deficit hyperactivity disorder (ADHD) versus untreated ADHD. Improvement was reported most often in studies of driving and obesity outcomes (left side), with a greater proportion of outcomes reported to exhibit no benefit following treatment compared with no treatment in studies of occupation (right side). An intermediate proportion of studies of self-esteem, social function, academic, drug use/addictive behavior, antisocial behavior, and services use outcomes reported benefit with treatment.

Benefit with treatment was analyzed according to specific outcome group for participants with treated versus untreated ADHD (Figure 6). Note that this analysis involved the same 48 studies and 76 outcomes in the analysis shown in Figure 5, with the same 3 conditions considered as beneficial with treatment and the same 3 considered to exhibit no benefit with treatment. For 100% of driving and obesity outcomes reported, treatment of ADHD was beneficial. For 90% of self-esteem outcomes, 83% of social function outcomes, 71% of academic outcomes, 67% of drug use/addictive behavior outcomes, 50% of antisocial behavior outcomes, 50% of services use outcomes and 33% of occupation outcomes, treatment was reported to be beneficial. In the case of services use, less use of services (for example, emergency room visits, financial assistance) was considered to be an improvement with treatment.

**Figure 7:**
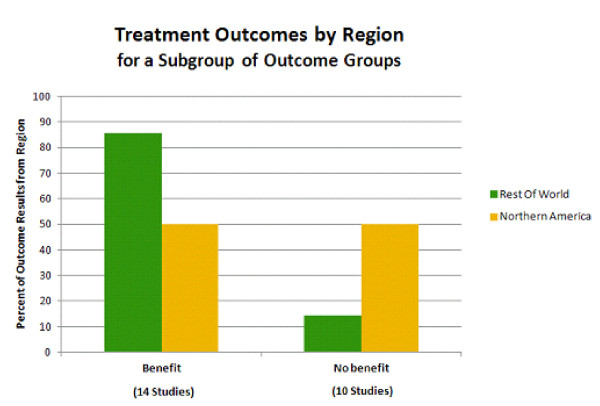
**Treatment results by region for a subgroup of outcomes**. Outcomes exhibiting benefit versus no benefit with treatment are shown for Northern America (yellow bars) versus the rest of the world (green bars). Note that Northern America includes Canada and the USA, and Rest of World, in this case, consists of countries in Europe. The response to treatment for four outcome groups was included: drug use/addictive behavior, antisocial behavior, services use, and occupation outcomes. The percentage of studies reporting benefit with treatment for these outcomes is greater for the rest of the world compared with Northern America. As in Figure 5, the sum of the numbers of studies shown under each bar does not equal the total number of studies of this type, because a single study reported an outcome that exhibited benefit from treatment and one that did not, and so this study is represented in both types of outcome.

Four of the nine outcome groups exhibited a substantial percentage of no benefit with treatment. These four outcome groups were drug use/addictive behavior, antisocial behavior, services use, and occupation. A subanalysis of these four outcomes examined the *post-hoc *hypothesis that less aggressive/consistent treatment of ADHD in the rest of the world compared with Northern America (based in part on stricter diagnosis criteria for the *International Classification of Diseases*, tenth edition (ICD-10) versus the DSM-IV-TR [49,50]) may account for the rate of reported treatment benefit observed in these four outcome groups in Figure 6. Thus, treatment outcome by region for this subgroup of outcomes was examined (Figure 7). For these 4 outcome groups, studies performed in Northern America were evenly split in reporting outcome results exhibiting treatment benefit versus no benefit (11 outcome results each, 50% each). In contrast, studies performed in countries in the rest of the world (all from Europe, in this case) reported a higher percentage of outcome results exhibiting treatment benefit (six of seven outcome results; 86%, four of which were improvement in drug use/addictive behavior outcomes) versus no benefit with treatment (one of seven outcome results; 14%) (Figure 7). This result clearly shows that reported results of treatment in the rest of the world do not underlie the higher percentage of outcome results exhibiting no benefit with treatment for these four outcomes. On the contrary, studies from the rest of the world reported a larger percentage of outcomes exhibiting treatment benefits.

Further analysis of the results observed in Figure 7 showed that this result did not appear to be associated with types of treatment, comparator groups, or outcome groups of interest in Europe compared with Northern America [35]. A greater percentage, however, of these outcome results from the rest of the world were reported in retrospective studies of adults (3 of 4 studies; 75%) compared with those from Northern America (2 of 19 studies; 11%). The majority of these outcomes from Northern America were reported in prospective studies of various age groups (15 of 19 studies; 79%). Thus, differences in study design may underlie the result shown in Figure 7. The various treatment types mentioned in all the studies are listed in Additional File 2. Of the 130 studies that mention treatment, 120 (92%), 49 (38%), and 24 (18%) studies mentioned pharmacological treatment, non-pharmacological treatment, and/or multimodal treatment, respectively.

## Discussion

Overall, the results of the present study show that the long-term outcomes for participants with ADHD when left untreated were poor compared with non-ADHD controls, and that treatment of ADHD improved long-term outcomes, but usually not to the point of normalization. The outcomes that were studied (with ADHD symptoms deliberately excluded as an outcome) most often included drug use/addictive behavior, academic, and antisocial behavior. This was followed by social function, self-esteem, occupation, driving, services use, and obesity outcomes. These trends may reflect what is of most immediate interest to society in a given time period. For example, obesity, the least-studied outcome, has come into interest only recently, likely due to the increasing obesity epidemic in developed countries. Increasing interest in the epidemiology of obesity, led to the report of an association between obesity and ADHD in 2002 [51]. Our data also indicate that there are specific geographical trends, with academic outcomes being of greater interest for study in the US and Canada and antisocial behaviors of greater interest in Europe. This difference of interest may be a function of only more severe cases, likely to have oppositional-defiant or conduct disorder comorbidity, being diagnosed outside Northern America. These trends have been described in more detail in a separate publication [35].

The number of studies of long-term outcomes of ADHD has risen noticeably over the last 30 years, especially since 2000. This corresponds to a trend in awareness of the consequences of ADHD by clinicians, which appears to be on the rise [52].

Treatment resulted in beneficial effects for many of the outcomes reported (72% of outcome results). These beneficial effects were observed as either significant improvement over pretreatment baseline, in comparison to untreated ADHD participants, or stabilization of the outcomes (that is, prevention of the deterioration over time from baseline reported with untreated ADHD [43-46]). Driving and obesity outcomes were the most often reported to be responsive to treatment. Of course, a decrease in obesity may be due to an appetite suppressant effect of stimulants and atomoxetine. The relatively small number of studies of these two outcomes (two studies each) comparing treated with untreated ADHD and the consistently positive response to treatment support further investigation in these areas. Three other outcomes that were often reported to be responsive to treatment were self-esteem, social function, and academic outcomes. These results are supported by a relatively large number of studies (10, 12, and 21 studies, respectively) comparing participants with treated ADHD with participants with untreated ADHD. These outcomes may be more closely related to symptom relief. The outcomes reported are not independent of one another and changes in one may reflect changes in others. The wider effects of response to treatment in these two areas may warrant further investigation.

The four remaining outcomes that appeared to be least responsive to treatment were drug use/addictive behavior, antisocial behavior, services use, and occupation, with 67%, 50%, 50%, and 33% of reported outcome results demonstrating a benefit of treatment, respectively. Persistence in these cases may have to do with the existence of comorbidities, such as conduct disorder, which has for example, been associated with increased substance use disorders [53]. Oppositional defiant disorder or conduct disorder, may contribute to long-term outcomes in people with ADHD, specifically crime and substance use [54-56], and thus may affect the response observed for these outcomes with treatment for ADHD. Other comorbidities, such as depression, obsessive-compulsive disorder, or autism may have similarly influenced the results we observed.

Services use may persist due to the incomplete amelioration of ADHD symptoms and impairments, possibly because although ADHD symptoms (like outcomes) respond to treatment, they are not completely normalized. Finally, continued impairment in occupation despite treatment may reflect the cumulative effects of ADHD symptoms and dysfunctioning over the lifespan. For example, low academic grades may later restrict employment or opportunities, impaired social function may precipitate extra friction with employers. The differential responsiveness of different outcomes to treatment is an intriguing area for future study.

It should be mentioned that if we had categorized the study outcomes by age, such as 5 to 17 and over 18, we may have observed different areas of improvement depending on the age group. In combining the groups it is possible that this distinction is lost. One also needs to consider however, that certain categories such as occupation would not be as relevant to the 5 to 17 age group as opposed to academic achievement, which would apply to all groups.

Even with treatment, worse outcomes were often reported for the ADHD group than for people without ADHD. This is not surprising, because although behavioral and drug treatment have been demonstrated to improve ADHD symptoms, these treatments do not necessarily normalize behavior to control levels [35,57-61].

For example, in one study of the effect of methylphenidate treatment on classroom measures, a 20 mg dose produced normalization in 30% to 60% of participants, (depending on the measurement used) although 53% to 94% showed improvement [58]. Many studies (42) in our analysis evaluated treatment effects only against non-ADHD controls, as opposed to pretreatment baseline or any untreated state. In these studies, only 24% of outcomes were reported to be similar for treated ADHD and non-ADHD controls. For all the other outcomes reported (76%), the outcomes remained worse than non-ADHD controls, and there was no mechanism with this study design by which to measure improvement with treatment that did not completely 'normalize' the outcome. In studies with other study designs (comparing participants with treated ADHD and participants with untreated ADHD or pretreatment baseline), benefit with treatment was reported for 72% of the outcomes. These study designs allowed the improvement with treatment to be demonstrated, even though the outcome may not have 'normalized'. When considering the effects of treatment reported in any one study, the comparator group used to evaluate the effectiveness of ADHD treatment is particularly important.

The results of four studies included in this analysis that used both types of comparators within the same study ((1) non-ADHD controls and (2) untreated ADHD participants or ADHD participant's pretreatment baseline) were consistent with the present overall observations that there was clear improvement or stabilization with treatment of ADHD for social function, antisocial behavior, and academic outcomes, but not to the extent that non-ADHD control outcomes were matched [13,39,48,62]. This general pattern was noticed in the earliest of the four studies, as the authors conclude in their 12-year follow-up study that 'The most striking finding of the study is the repetitive pattern of finding significant differences between the stimulant-treated hyperactives and their control group (with the control group almost invariably doing better). However, there are several areas in which the stimulant-treated hyperactives seem to do better than their untreated counterparts' (referring to academic, driving, self-esteem, and social function outcomes) [13]. This pattern was also found in an 8-year follow-up study of a different sample 'despite overall maintenance of improvement in functioning relative to baseline (pretreatment), the MTA group as a whole was functioning significantly less well than the non-ADHD classmate sample' [39]. In the present study, this pattern was broadly replicated across the outcome groups when analyzed individually. Comparisons against non-ADHD controls only, may mask improvements with treatment.

It should be noted that ADHD Rating Scale IV total scores decline (improve) between ages 5 to 7 and ages 14 to 18 in both Caucasians and African-Americans (although in Latinos they actually increase during these time periods). Hyperactivity-impulsivity scores, especially, decline from ages 4 to 7 to age 14 and older in both boys and girls [63]. Therefore, we cannot discount that natural decline in symptoms (and possibly other dysfunctions) occurring over time also contributes to the improvement in outcomes observed with treatment, although this idea contradicts the reported worsening of functional domains in untreated ADHD. According to one paper, symptoms and functioning are related. With full symptom remission, illicit drug use and antisocial behaviors become similar to non-ADHD controls, but while social function improves, it does not reach non-ADHD levels [64].

Finally, we observed that treatment outcomes for the subgroup of domains that exhibit lower percentages of outcomes that benefit from treatment (drug use/addictive behavior, antisocial behavior, services use, and occupation) were differentially improved when studies from Northern America were compared with those from the rest of the world (in this case, all 'Rest of World' studies were from Europe). Based on this analysis there appears to be a geographical bias with regard to how responsive these four outcome group results are reported to be with treatment, a result that may be accounted for by regional differences in study design along with the resulting study population age, or diagnostic practices. In this very specific comparison, the numbers of studies from 'Rest of World' countries are low (four studies, seven outcome results), thus as further investigation of these outcomes around the world are published, the results of this comparison may be clarified.

### Limitations and possible sources of bias

Several possible risks of bias and limitations need to be considered regarding the included studies. First, a publication and cultural bias could have resulted from including only studies that were published in English. In addition, the analysis excluded unpublished studies that might have been presented at conferences, for example. Also, our search relied on search engines for 'peer reviewed' status. Moreover, by strictly adhering to Cochrane systematic review guidelines and only including studies that were identified in our original electronic search, it is possible that some relevant studies may have been missed, introducing a search engine and literature database bias. This bias was reduced by extensive searching of 12 databases. Nonetheless, we are aware of four studies that would have met inclusion criteria, but were not identified by the search engines due to a technical limitation or inadvertent search string exclusion. Examination of these studies shows that the reported results are consistent with the overall results of the present analysis. The results of these four studies are summarized briefly here. One study reported poorer outcomes for participants with ADHD versus non-ADHD controls with regard to academic achievement, occupational adjustment, antisocial behavior, relationships, and substance use [65]. A second study found a high incidence of ADHD (65%) in 23 adolescents who attempted suicide [66]. A third study reported that by young adulthood, participants with ADHD were similar to non-ADHD controls in minor aspects of social and occupational outcomes (e.g., time socializing with friends and with hobbies), but had poorer outcomes in major aspects of these outcomes (e.g., had many more offspring and most were not living with them) [67]. A fourth study found that stimulant treatment in children with ADHD significantly improved reading scores and decreased grade retention [68]. The results of these studies are consistent with our overall finding that untreated ADHD is associated with poor long-term outcomes and that these outcomes improve with treatment.

Researcher bias could also be a possible source of bias in this analysis, however, this was reduced by having two researchers independently agree on the articles included and strict, simple inclusion criteria were established prior to searching.

Other sources of bias could include biases of individual research groups, which was eliminated by including only electronically identified studies (as mentioned above) and not selectively including the studies of specific groups and omitting others. As observed, different study designs may also lead to different conclusions and taking comparators into consideration is critical. We included studies of various designs, which may minimize such bias.

A further possible bias could arise from changes over time in diagnostic criteria or discrepancy between classification systems, specifically differences in the definition of hyperkinetic disorder (ICD-9 or ICD-10) versus ADHD (DSM-III/DSM-III-R/DSM-IV). Differences are less likely within classification systems [3]. One study found that 93% of children diagnosed with ADHD using DSM-III-R diagnosis also received a DSM-IV ADHD diagnosis, indicating good correspondence between classification systems [69]. Rediagnosis of the MTA sample by ICD criteria, however, resulted in only 25% of the DSM-IV-diagnosed MTA sample of combined-type ADHD qualifying as having hyperkinetic disorder or hyperkinetic conduct disorder by ICD-10 criteria [69].

## Conclusions

The present analysis supports the premise that without treatment, people with ADHD often experience poorer long-term outcomes and that treatment may improve the long-term outcomes of ADHD for some individuals, but not necessarily to the degree of healthy controls. Further analyses of the present data set will more comprehensively examine the impact of treatment on specific outcomes, as well as the impact of specific types of treatment modalities. The question remains as to whether the short-term benefits demonstrated by short-term drug or non-pharmacological treatment studies translate directly into long-term outcomes. Associations between specific short-term symptoms need to be examined as possible predictors for long-term outcomes, particularly because long-term studies are not always feasible. Future research should focus on the association between short-term symptom relief and long-term consequences and include longer-term follow-up of the consequences of childhood ADHD into the adult years.

## Competing interests

MS was an employee of Shire Pharmaceuticals, Ltd. when this analysis was conducted and when this manuscript was drafted. PH is an employee of Shire Development LLC, owns Shire stock and has stock options. HC has received consulting fees from Shire (nothing for contributing to this article). SY has received research funding or consulting fees from Janssen-Cilag, Eli-Lilly, Novatis, Flynn-Pharma and Shire (nothing for contributing to this article). She was a member of the UK NICE Guideline Development Group for ADHD. JK is owner of BPS International. BPS International received funding from Shire Development LLC to perform this analysis and participate in writing this manuscript. AGW is a consultant for BPS International and has been a consultant for Shire Development LLC. LEA has received research funding or consulting fees from Astrazeneca, Biomarin, Curemark, Lilly, Novartis, Noven, Seaside Therapeutics, and Shire (nothing for contributing to this article). Funded by Shire Development LLC, Wayne PA. An earlier version of this analysis was presented as a poster [70].

## Authors' contributions

All authors participated in designing and interpreting this analysis. JK, MS, and AW oversaw the literature search and analysis. All authors participated in writing portions of this manuscript and in reviewing and approving all manuscript drafts.

## Authors' information

MS was previously International Medical Director Clinical Development at Shire Pharmaceuticals and is currently Development Therapy Area Director at Norgine Pharmaceuticals. PH is Senior Director Global Health Economics & Outcomes Research at Shire Development LLC. HC is a Child and Adolescent Psychiatrist at Hôpitaux Pédiatriques de Nice CHU Lenval. SY is a Senior Lecturer in Forensic Clinical Psychology at King's College London, Institute of Psychiatry. JK is Founder and Chief Editor at BPS, International in San Diego, California. AGW is a consultant for BPS International, was formerly a consultant for Shire Development LLC, and is currently a Research Assistant Professor of Chemistry and Biomolecular Science, at Clarkson University in Potsdam, New York. LEA is a Child Psychiatrist, Educator, Professor Emeritus of Psychiatry, and a Principal Investigator in the Psychopharmacology and Related Research Program at the Ohio State University Nisonger Center.

## Pre-publication history

The pre-publication history for this paper can be accessed here:

http://www.biomedcentral.com/1741-7015/10/99/prepub

## Supplementary Material

Additional file 1 **Search strategy details**. These are the specific details of the search strategy used in this systematic review. Click here for file

Additional file 2 **Treatment types reported in the included studies**. This list includes all the treatments mentioned in any study. Often a treatment may have been listed in the Methods of a study but no details were provided about dose or duration or age of treatment or frequency of treatment or separate connection to a specific outcome result, for example. It was possible to group treatment types by large category (pharmacological, non-pharmacological, or MMT) and pool the reported outcomes in these categories. Click here for file
