# Supplementary material for: A systematic review and analysis of long-term outcomes in attention deficit hyperactivity disorder: effects of treatment and non-treatment
Source: BMC Med. 2012 Sep 4;10:99. doi: 10.1186/1741-7015-10-99 (PMC3520745; doi:10.1186/1741-7015-10-99)
Supplement: Additional file 1 — Search strategy details. These are the specific details of the search strategy used in this systematic review. [file 1741-7015-10-99-S1.DOC]

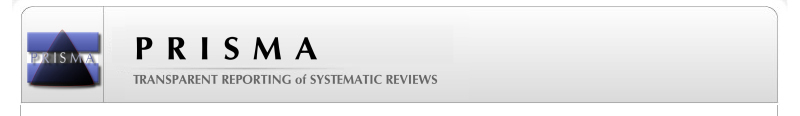
**PRISMA 2009 Flow Diagram**

**Screening**

**Included**

**Eligibility**

**Identification**

Records identified through database searching
(n = 7272)

Additional records identified through other sources
(n = 0)

Records after duplicates removed
(n = 5467)

Records screened
(n = 5467)

Records excluded
(n = 4818)

Full-text articles assessed for eligibility
(n = 649)

Full-text articles excluded, with reasons
(n = 298)

Studies included in qualitative synthesis
(n = 351)

1. The following databases were searched using the search strategy copied below. Academic Search Premier, CINAHL, Cochrane Central Register of Controlled Trials (includes EMBASE), ERIC, MEDLINE, Military & Government collection, NHS Economic Evaluation database, PsycARTICLES, PsycINFO, SocINDEX with Full Text, and Teacher Reference Center.

The actual search strategy and selected limiters copy/pasted from the search engine window follows.

(SU attention deficit disorder or SU hyperkine* or SU “TDAH” or SU “DAH” or SU “DAA”) and (TX "longterm" or TX "long term" or TX education or TX academic or TX degree or TX school or TX grade or TX promotion or TX "IQ" or TX “standardized test scores” or TX “standardised test scores” or TX detention or TX expulsion or TX graduation or TX activities or TX sports or TX hobbies or TX socioeconomic or TX salary or TX “work productivity” or TX “occupational injury” or TX functional or TX divorce or TX relations* or TX criminal* or TX incarcerat* or TX arrest or TX automobile or TX car or TX driving or TX citation* or TX crash* or TX weight or TX height or TX obesity or TX “drug abuse” or TX addiction or TX “substance abuse” or TX “illegal substance use” or TX “illicit substance use” or TX alcoholi* or TX “alcohol abuse” or TX suicid*) not SU neuroanatom* not SU neuropatholog* not SU patholog* not SU pathophysiology not SU molecular not SU gene not SU genetic* not SU genotype not SU development* not SU validity not SU “differential diagnosis” not SU SPECT not SU electromyography not SU “frontal lobe” not SU “reaction time” not SU “response time” not SU "chemically induced" not SU physiopathology not SU etiology not SU aetiology not SU preclinical not SU "Phase I" not SU "Phase II" not SU tolerability not SU pharmaco* not SU dose-finding not SU modeling not SU modelling not SU ethics not SU reprint not SU “conference presentation” not SU “literature review” not PT review not PT supplement and (TX control or TX proband* or TX normal or TX placebo or TX untreated or TX “no treatment” or TX nonmedication or TX “non medication” or TX unmedicated or TX “no therapy” or TX compar* or TX premedication or TX pretreatment or TX “pre treatment” or TX longitudinal or TX retrospective or TX followup or TX “follow up” or TX “standard care”)

Limiters - Scholarly (Peer Reviewed) Journals; Published Date from: 19800101-20101231; English Language; Human; Age Related: Child, Preschool: 2-5 years, Child: 6-12 years, Adolescent: 13-18 years, Young Adult: 19-24 years, Adult: 19-44 years, Middle Aged: 45-64 years, Middle Aged + Aged: 45+ years, Aged: 65+ years, Aged, 80 and over, All Adult: 19+ years; Publication Type: Clinical Trial, Clinical Trial, Phase III, Clinical Trial, Phase IV, Controlled Clinical Trial, English Abstract, Government Publications, Introductory Journal Article, Journal Article, Multicenter Study, Published Erratum, Randomized Controlled Trial, Twin Study; Languages: English; Publication Type: Periodical, Book, Primary Source Document; Document Type: Article, Book Chapter, Erratum, Report; Publication Year from: 1980-2010; English Language; Publication Type: Book Chapter, Clinical Trial, Corrected Article, Journal Article, Research; Language: English; Age Groups: Child, Preschool: 2-5 years, Child: 6-12 years, Adolescent: 13-18 years, Adult: 19-44 years, Middle Aged: 45-64 years, Aged: 65+ years, Aged, 80 and over; MEDLINE Publication Type: Multicenter Study, Published Erratum, Randomized Controlled Trial, Twin Study, Clinical Trial, Clinical Trial, Phase III, Clinical Trial, Phase IV, Controlled Clinical Trial, Corrected and Republished Article, Journal Article; Publication Type: Books, Journal Articles, Reports (All); Language: English; Publication Type: Primary Source Document, Periodical, Government Document; Date Abstract Published from: 1980-2010; Year of Publication - Reviews from: 1980-2010; Type of Intervention: Treatment, Management, Rehabilitation, Palliative Treatment, Education, Supportive Care, Disease Management; Year of Publication from: 1980-2010; Exclude Book Reviews; Exclude Non-Article Content; Age Groups: Preschool Age (2-5 years), School Age (6-12 years), Adolescence (13-17 years), Adulthood (18 years & older), Young Adulthood (18-29 years), Thirties (30-39 years), Middle Age (40-64 years), Aged (65 years & older), Very Old (85 years & older); Population Group: Human; Methodology: EMPIRICAL STUDY, FIELD STUDY, TREATMENT OUTCOME/CLINICAL TRIAL; Document Type: Erratum/Correction; Publication Year from: 1980-2010; Publication Type: Peer Reviewed Journal; English; Language: English; Age Groups: Preschool Age (2-5 years), School Age (6-12 years), Adolescence (13-17 years), Adulthood (18 years & older), Young Adulthood (18-29 years), Thirties (30-39 years), Middle Age (40-64 years), Aged (65 years & older), Very Old (85 years & older); Population Group: Human; Document Type: Chapter, Erratum/Correction, Journal Article; Methodology: EMPIRICAL STUDY, FIELD STUDY, TREATMENT OUTCOME/CLINICAL TRIAL; Exclude Dissertations; Publication Type: Periodical, Book; Document Type: Article, Book Chapter, Erratum, Report

Search modes - Boolean/Phrase

2. A second search of MEDLINE was performed using the PubMed search engine, using the same search strategy and limiters.

The actual search strategy and limiters copy/pasted from the PubMed search engine window follows. (attention deficit disorder [MeSH Terms] OR hyperkine* [MeSH Terms] OR "DAH" [MeSH Terms] OR "TDAH" [MeSH Terms] OR “DAA” [MeSH Terms]) AND ("longterm" [TW] OR "long term" [TW] OR education [TW] OR academic [TW] OR degree [TW] OR school [TW] OR grade [TW] OR promotion [TW] OR "IQ" [TW] OR "standardized test scores" [TW] OR detention [TW] OR expulsion [TW] OR graduation [TW] OR activities [TW] OR sports [TW] OR hobbies [TW] OR socioeconomic [TW] OR salary [TW] OR "work productivity" [TW] OR "occupational injury" [TW] OR functional [TW] OR divorce [TW] OR relations* [TW] OR criminal* [TW] OR incarcerat* [TW] OR arrest [TW] OR automobile [TW] OR car [TW] OR driving [TW] OR citation* [TW] OR crash* [TW] OR weight [TW] OR height [TW] OR obesity [TW] OR "drug abuse" [TW] OR addiction [TW] OR "substance abuse" [TW] OR "illegal substance use" [TW] OR "illicit substance use" [TW] OR alcoholi* [TW] OR "alcohol abuse" [TW] OR suicid* [TW]) AND (control [TW] OR proband* [TW] OR normal [TW] OR placebo [TW] OR untreated [TW] OR "no treatment" [TW] OR nonmedicat* [TW] OR "non medicat*" [TW] OR unmedicated [TW] OR "no therapy" [TW] OR compar* [TW] OR premedication [TW] OR pretreatment [TW] OR "pre treatment" [TW] OR longitudinal [TW] OR retrospective [TW] OR followup [TW] OR "follow up" [TW] OR "standard care" [TW]) NOT neuroanatom* [MeSH Terms] NOT neuropatholog* [MeSH Terms] NOT patholog* [MeSH Terms] NOT pathophysiology [MeSH Terms] NOT molecular [MeSH Terms] NOT gene [MeSH Terms] NOT genetic* [MeSH Terms] NOT genotype [MeSH Terms] NOT developmental biology [MeSH Terms] NOT Reproducibility of Results [MeSH Terms] NOT "differential diagnosis" [MeSH Terms] NOT "Nuclear Magnetic Resonance, Biomolecular" [MeSH Terms] NOT electromyography [MeSH Terms] NOT "magnetic resonance imaging" [MeSH Terms] NOT "frontal lobe" [MeSH Terms] NOT "reaction time" [MeSH Terms] NOT "response time" [MeSH Terms] NOT "chemically induced" [MeSH Terms] NOT physiopathology [MeSH Terms] NOT etiology [MeSH Terms] NOT aetiology [MeSH Terms] NOT preclinical [MeSH Terms] NOT "Phase I" [MeSH Terms] NOT "Phase II" [MeSH Terms] NOT drug safety [MeSH Terms] NOT pharmacokinetics [MeSH Terms] NOT pharmacodynamics [MeSH Terms] NOT pharmacoeconomics [MeSH Terms] NOT dose-finding [MeSH Terms] NOT modeling [MeSH Terms] NOT modelling [MeSH Terms] NOT ethics [MeSH Terms] NOT reprint [PT] NOT "conference presentation" [PT] NOT "literature review" [PT] NOT review [PT] NOT supplement [PT] AND (Humans[Mesh] AND (Clinical Trial[ptyp] OR Randomized Controlled Trial[ptyp] OR Clinical Trial, Phase III[ptyp] OR Clinical Trial, Phase IV[ptyp] OR Controlled Clinical Trial[ptyp] OR Corrected and Republished Article[ptyp] OR English Abstract[ptyp] OR Government Publications[ptyp] OR Introductory Journal Article[ptyp] OR Journal Article[ptyp] OR Multicenter Study[ptyp] OR Twin Study[ptyp]) AND English[lang] AND medline[sb] AND (adult[MeSH] OR child, preschool[MeSH] OR child[MeSH:noexp] OR adolescent[MeSH] OR young adult[MeSH] OR adult[MeSH:noexp] OR middle age[MeSH] OR (middle age[MeSH] OR aged[MeSH]) OR aged[MeSH] OR aged, 80 and over[MeSH]) AND ("1980"[PDat] : "2010"[PDat]))

Limiters: Humans, Clinical Trial, Randomized Controlled Trial, Clinical Trial, Phase III, Clinical Trial, Phase IV, Controlled Clinical Trial, Corrected and Republished Article, English Abstract, Government Publications, Introductory Journal Article, Journal Article, Multicenter Study, Twin Study, English, MEDLINE, All Adult: 19+ years, Preschool Child: 2-5 years, Child: 6-12 years, Adolescent: 13-18 years, Young Adult: 19-24 years, Adult: 19-44 years, Middle Aged: 45-64 years, Middle Aged + Aged: 45+ years, Aged: 65+ years, 80 and over: 80+ years, Publication Date from 1980 to 2010

3. An additional broad search of attention deficit hyperactivity disorder (ADHD) in the Criminal Justice Abstracts was performed as follows. The search was limited to last updated English-language journal articles in which ADHD (and associated terms) were mentioned in the title, abstract, or descriptors.

TI ( (attention deficit disorder) or hyperkine* or “TDAH” or “DAH” or “DAA”) or AB ( (attention deficit disorder) or hyperkine* or “TDAH” or “DAH” or “DAA”) or KW ( (attention deficit disorder) or hyperkine* or “TDAH” or “DAH” or “DAA”)

Limiters - Scholarly (Peer Reviewed) Journals; Publication Date: 19800101-20101231; Publication Type: Academic Journal, Book, Periodical; Document Type: Article, Book Chapter, Erratum, Report

Search modes - Boolean/Phrase
