# Supplementary material for: A systematic review and analysis of long-term outcomes in attention deficit hyperactivity disorder: effects of treatment and non-treatment
Source: BMC Med. 2012 Sep 4;10:99. doi: 10.1186/1741-7015-10-99 (PMC3520745; doi:10.1186/1741-7015-10-99)
Supplement: Additional file 2 — Treatment types reported in the included studies. This list includes all the treatments mentioned in any study. Often a treatment may have been listed in the Methods of a study but no details were provided about dose or duration or age of treatment or frequency of treatment or separate connection to a specific outcome result, for example. It was possible to group treatment types by large category (pharmacological, non-pharmacological, or MMT) and pool the reported outcomes in these categories. [file 1741-7015-10-99-S2.DOC]

Treatment types reported in the included studies.

Pharmacological Treatment

Stimulants (short-acting or long-acting)

methylphenidate (Ritalin, Ritalin-LA, Concerta)

amphetamine sulfate, dextroamphetamine, dexamfetamine, *d*-amphetamine, (Dexadrine), lisdexamphetamine (Vyvanse)

mixed amphetamine salts (Adderall, Adderall XR)

pemoline; sometimes spelled premoline (Cylert)

Non-stimulants

atomoxetine (Strattera)

guanfacine (Intuniv)

reboxetine

clonidine

Other medication, including non-attention deficit hyperactivity disorder (ADHD) intended pharmaceuticals

Tricyclics, including imipramine

bupropion

fluoxetine

sertraline

citalopram

chlorpromazine

phenothiazines

Treatment (not specified, infer primarily pharmacological)

Non-pharmacological Treatment

Training and Therapies

behavioral therapy

psychotherapy

family therapy

educational therapy

occupational therapy

mixed therapy (drama, play, pet, art)

counseling

academic training

teacher training

parent training

social training

attention training

organizational skills training

Structured mentoring

academic mentoring

parent mentoring

Diet

Biofeedback

Multimodal Treatment: any combination of above treatments including at least one each of a pharmacological and non-pharmacological treatment.
